# Comparing dextrose prolotherapy with other substances in knee osteoarthritis pain relief: A systematic review

**DOI:** 10.1016/j.clinsp.2022.100037

**Published:** 2022-05-17

**Authors:** Vitor Santos Cortez, Walter Augusto Moraes, João Victor Taba, Alberto Condi, Milena Oliveira Suzuki, Fernanda Sayuri do Nascimento, Leonardo Zumerkorn Pipek, Vitoria Carneiro de Mattos, Matheus Belloni Torsani, Alberto Meyer, Wu Tu Hsing, Leandro Ryuchi Iuamoto

**Affiliations:** a Faculdade de Medicina, Universidade de São Paulo (FMUSP), São Paulo, SP, Brazil; b Department of Gastroenterology, Hospital das Clínicas, Faculdade de Medicina, Universidade de São Paulo (HCFMUSP), São Paulo, SP, Brazil; c Center of Acupuncture, Department of Orthopaedics and Traumatology, Universidade de São Paulo, São Paulo, SP, Brazil

**Keywords:** Prolotherapy, Dextrose, Knee, Osteoarthritis, Pain

## Abstract

The objective of this study is to compare the effectiveness of dextrose-prolotherapy with other substances for pain relief in patients with primary knee osteoarthritis. The literature screening was done in January 2021 through Medline (PubMed), EMBASE, and Database of the National Institute of Health based on the following criteria: randomized clinical trials that subjected patients with primary knee osteoarthritis who underwent treatment with dextrose-prolotherapy and other substances for pain relief. Paired reviewers independently identified 3381 articles and included 8 trials that met the eligibility criteria. According to the findings of this review, participants that underwent dextrose-prolotherapy showed improvements between baseline and posterior assessments and when compared to saline injections, but when compared to other substances, the results were not clear. Although dextrose-prolotherapy is a useful treatment method by itself, it is still not possible to clearly affirm that it is superior or inferior to its counterparts. There is an urgent need for further studies to bring more evidence to the field.

## Highlights

• Dextrose injections promote deposition of collagen into injured structures through growth factors and inflammatory cells.
• Dextrose-prolotherapy is a useful treatment method, but it is not superior or inferior to its counterparts.

## Introduction

Knee Osteoarthritis (KOA) is a degenerative disease of the knee joint that causes pain and restricted range of motion, often impairing the patient's quality of life. It is a major medical condition thought to affect over 600 million people worldwide, with a prevalence of 22.9% in individuals aged 40 or above.^1^^,^^2^ A person diagnosed with KOA is expected to spend over $140,300 in treatment during the course of his/her life.^1^ In the USA, the annual costs attributed to osteoarthritis sit between 400 and 500 billion dollars, and these are expected to increase in the next few years.^3^ Unfortunately, even with a vast array of treatment options, failed attempts and refractory symptoms still appear to be very prevalent.^1^, ^2^, ^3^

Research on D-PRL treatment for KOA has increased significantly over the last 10 years, thus signaling the need for a new analysis of its efficacy as a pain relief method when compared to other therapies.^4^ Though its precise mechanisms are still debated, it has been hypothesized that intra-articular dextrose injections promote a regional influx of growth factors and inflammatory cells that ultimately provoke the deposition of new collagen into injured structures.^5^^,^^6^

Arias-Vázquez et al.^4^ sought to compare the usage of D-PRL with saline solution, HA, ozone infiltration, PRP, erythropoietin, and radiofrequency recently. However, this review potentially missed valuable studies in its analysis by limiting the literature search timeframe from January 2000 to May 2018 and the database search to PubMed, SciELO, and Google Scholar. Additionally, it is important to follow the AMSTAR-2 guidelines. This lack of methodological rigor might not ensure unbiased results and, therefore, a new review is needed.

The present study's aim is to develop a systematic review of the literature to compare the effectiveness of D-PRL with other substances for pain relief in patients with primary knee osteoarthritis.

## Material and methods

This systematic review was carried out in accordance with the items of Preferred Reports for Systematic Reviews and Protocol Meta-Analysis (PRISMA-P)^7^ and Assessing the Methodological Quality of Systematic Reviews (AMSTAR-2) guidelines.^8^ This study was registered by the Prospective Register of Systematic Reviews (PROSPERO, identification code CRD42021243755) before the research was carried out.

Drafting of the research question was based on the PICO strategy^9^ considering: patients with primary knee osteoarthritis (Patient or Problem); dextrose prolotherapy (Intervention or Assessment); other substances comparison (Control or Comparison); pain relief outcomes available in the literature were considered in the analysis (Outcome).

### Eligibility criteria

#### Inclusion criteria

##### Types of studies

Only Randomized Clinical Trials (RCT) were considered, and the articles were selected from their titles and abstracts according to their data relevance and regardless of their publication status.

RCTs were favored over other types of studies due to their capability of producing high-quality evidence, given that the goal of this review was to compare the efficacy of clinical interventions.

##### Types of participants

Study participants were patients with primary KOA who underwent treatment with dextrose prolotherapy and other substances for pain relief.

##### Types of intervention

The studies included must have had an intervention group that was treated with D-PRL. Control groups were characterized by either treatment with placebo or with therapeutic medical interventions other than D-PRL. Interventions that were common to all groups within a study were also allowed into the present review.

### Exclusion criteria

Studies will be excluded if: (1) Do not use a standard assessment method for the entire duration of the study or do not have pre-assessment; (2) Use dextrose prolotherapy as a single evaluation method or in a control group; (3) Compare dextrose prolotherapy only to non-interventional treatments; (4) Are not related to the question in the review; (5) Are in a language other than English, Portuguese or Spanish; (6) Are incomplete, unpublished or inaccessible articles to the authors.

#### Types of variables/parameters analyzed

Data were collected regarding the authors, date, and country of publication, the number of participants analyzed, sex, age, body mass index, KOA grade, group design, time of interventions and assessments, main parameters analyzed, main results, conclusions, funding and reported limitations of each study.

### Literature revision

The survey was conducted on January 25, 2021, without language restrictions, in the Medline database (via PubMed), EMBASE, and Database of the National Institute of Health (NIH).

Using the search tool, the authors selected MeSH terms from the most relevant publications to conduct a new search in order to obtain articles that could be included in this systematic review. In addition, a manual search of theses, meetings, references, study records, and contact with experts in the field was carried out.

### Search strategy

The same keywords were used in all databases, respecting their heterogeneities (for example, Emtree terms and MeSH terms were mapped in Embase and Medline, respectively).

The search strategy was: ((Prolotherapy) OR (Dextrose) OR (glucose) OR (injection)) AND ((Knee) OR (patellar)) AND ((osteoarthritis*) OR (osteo-arthritis*) OR (Osteoarthrotic) OR (Osteoarthrosis*) OR (arthralgia) OR (degenerate*) OR (Degenerative joint disease) OR (gonarthrosis)) AND ((Pain Management) OR (Pain) OR (Chronic Pain)).

### Data extraction

The data for each study were extracted independently by three authors (VSC, JVT, and WAM). Disagreements were resolved by consensus. If no consensus was reached, a fourth author (AM) would be consulted. Data extraction was carried out using the Rayyan tool ‒ https://rayyan.qcri.org/.^10^

All studies were analyzed according to their titles and abstracts, according to inclusion and exclusion criteria. If the eligibility criteria were met, the full text would be extracted. All studies eligible for qualitative analysis were described in the ""Results"" section.

Missing data were clarified by contacting the authors directly.

### Data validation

Four authors (VSC, WAM, JVT, and AC) carried out the data validation through the discussion of the selected works. If no consensus was reached, a fifth author (LI) would be consulted.

The risk of bias for intervention-type studies was analyzed using the guidelines of the Cochrane Back Review Group (CBRG).^11^

### Statistical analysis

If sufficient studies with a satisfactory quality were available, a meta-analysis would have been carried out with measures of heterogeneity and publication bias. Unfortunately, due to the heterogeneity of the data between eligible studies, no proper statistical analysis could be performed.

## Results

### Research flow

**Fig. 1:**
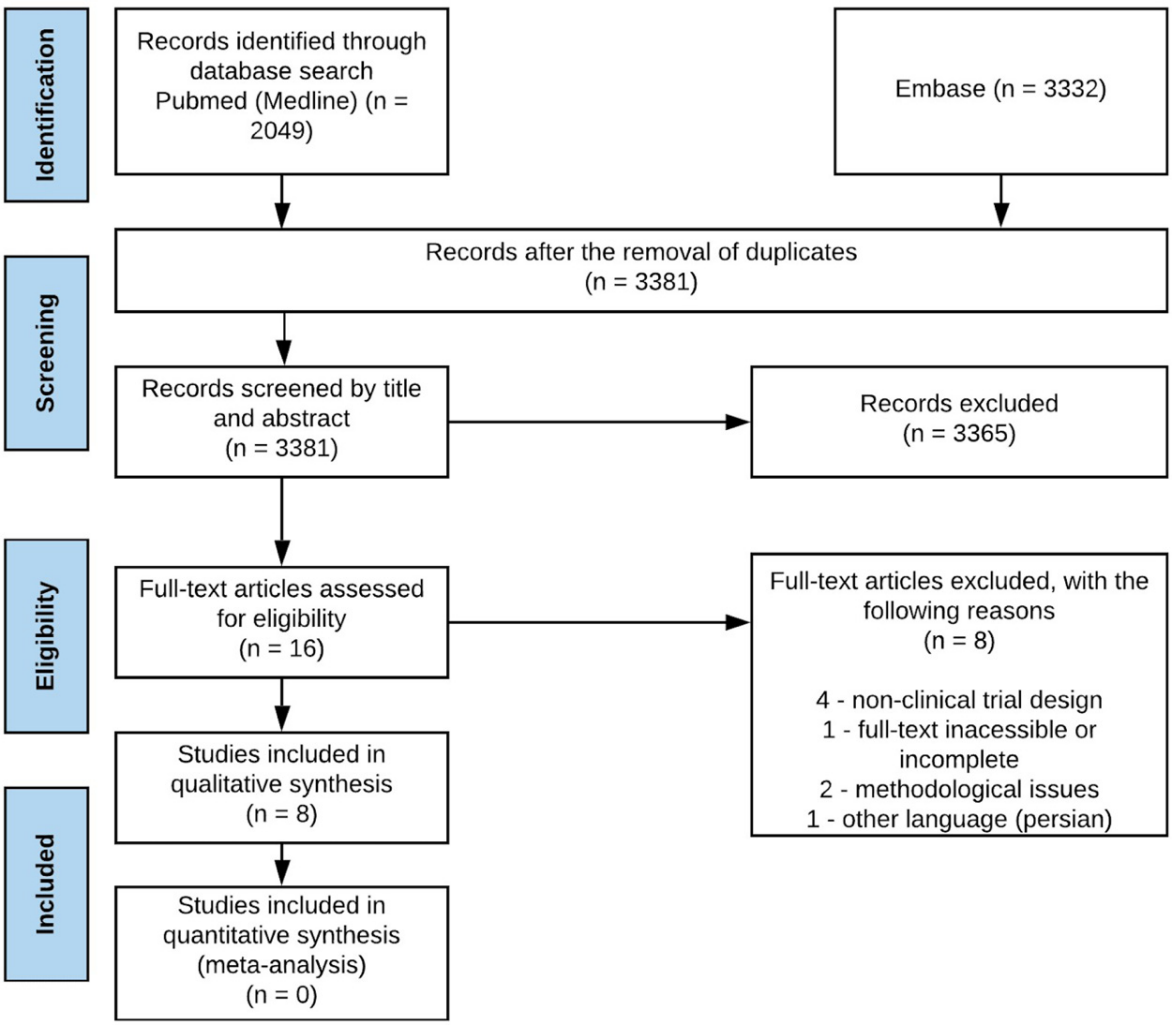
Research flow.

The electronic search found 5381 results for the keywords used. After removing 2000 duplicates and screening through abstract, the authors considered 16 potentially eligible studies for full-text analysis. Of these, 8 did not respect the exclusion criteria. Only 8 studies were considered eligible for qualitative analysis (Fig. 1).

### Quality of evidence

**Fig. 2:**
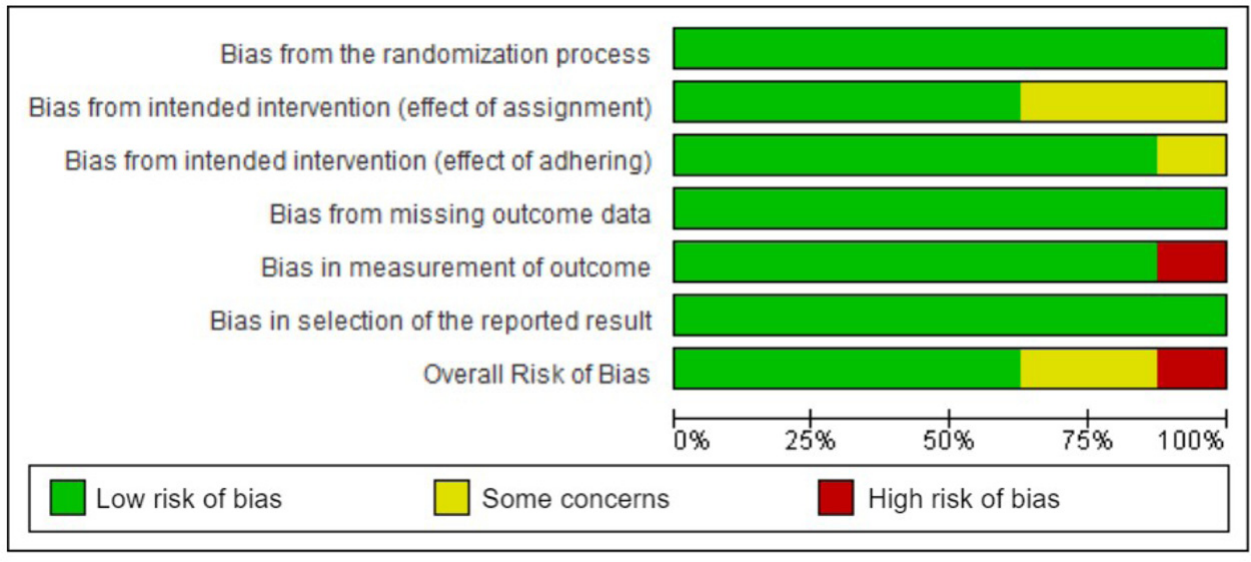
Graph of risk analysis of general bias in articles.

**Fig. 3:**
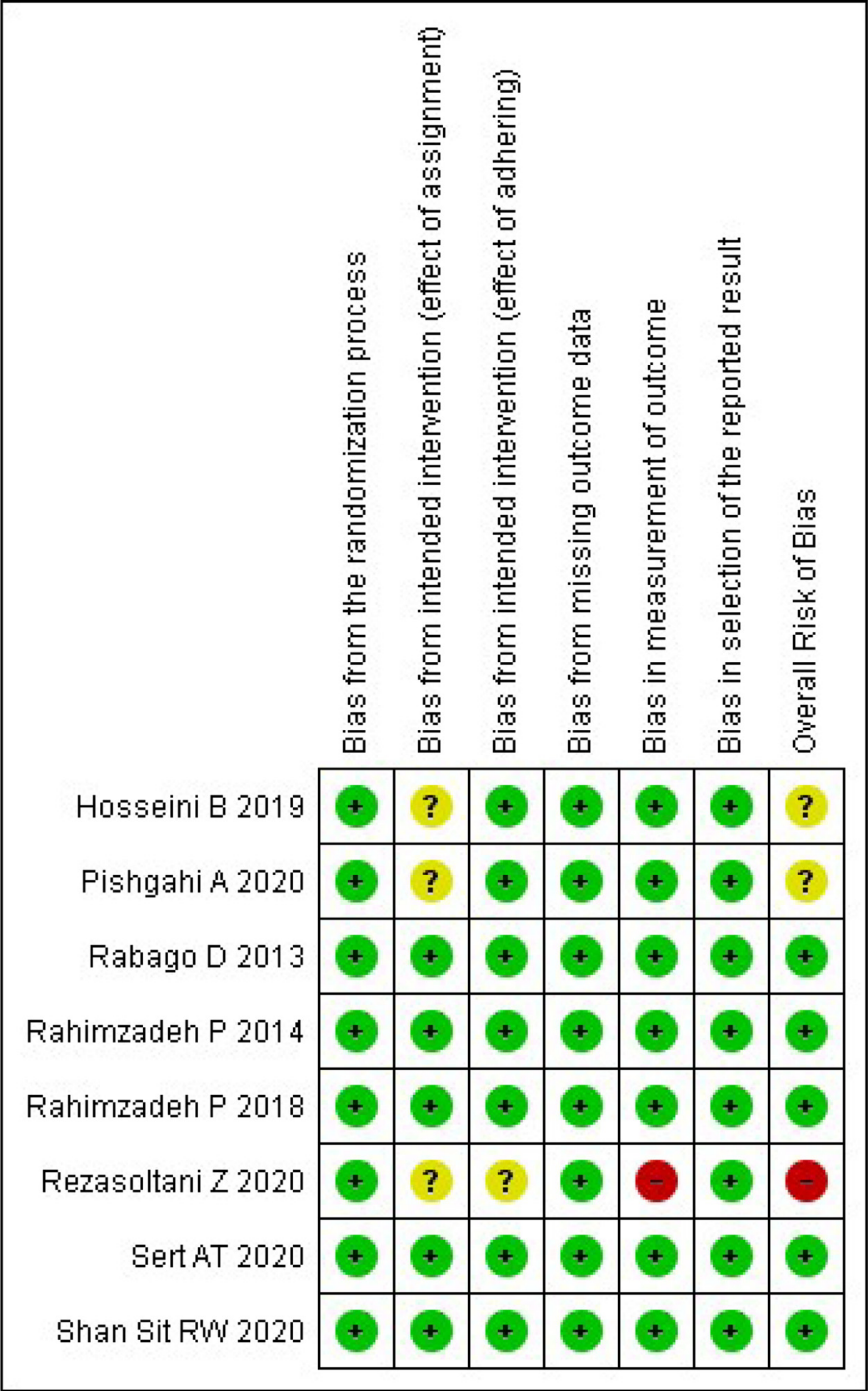
Summary of risk analysis of general articles bias.

After reading the articles included in the systematic review, the following factors were analyzed to determine the level of evidence: randomization process, intended intervention (effect of assignment and adhering), missing outcome data, measurement of outcomes, and reported results. The summary of the risk of bias analysis for each of the included articles is shown in Fig. 2, Fig. 3.

A total of 5 articles were classified as having a low overall risk of bias, Rabago et al.^12^ Rahimzadeh et al.^13^^,^^14^ Sert et al.^15^ and Shan Sit et al.^16^ The remaining 3, Rezasoltani et al.^17^ Hosseini et al.^18^ and Pishgahi et al.^19^ did not fall in this category, the first being high risk, whilst the other two were in some concerns category.^12^, ^13^, ^14^, ^15^, ^16^, ^17^, ^18^, ^19^

All articles achieved a low risk of bias in the categories of the randomization process, selection of the reported results, and missing outcome data. Therefore, they met the determined criteria successfully with a randomized and concealed allocation sequence, a proper data analysis with a pre-specified plan, and all outcome data being disclosed.

Regarding the ""Bias from intended intervention (effect of assignment")", Hosseini et al.^18^ Rezasoltani et al.^17^ and Pishgahi et al.^19^ were determined to be on the ""some concerns"" rank, due to the lack of disclosure that would allow us to properly assign them a ""low bias"".^17^, ^18^, ^19^ The remainder did accomplish all the expected requirements.

Rezasoltani et al.^17^ was the only article classified as a non-low risk in the other three categories, being in the ""some concerns"" rank in ""Bias from the intended intervention (effect of adhering")" and ""Bias in the measurement of outcome"", because he did not design a double-blinded study, as he thought it would not be possible considering the nature of such intervention, and a lacked disclosure of relevant information concerning parts of his methodology.

### Study characteristics

All included studies are complete, published, and have no conflict of interest. Doubts about the available data were supplemented by contacting the respective authors. The demographic characteristics collected are shown in Table 1; the methodological characteristics are shown in Table 2; the main results and conclusions are available in Table 3.

**Table 1:** Demographic characteristics of the studies.

Author (publication year, country)	Initial number of participants	Mean age (years) (SD)	Sex (male/female)	Mean BMI (kg/m²) (SD)	KOA grade^a^
Delgado et al.^22^	Total: 42	D-PRL: 64.3 ± 5.31	D-PRL: 11/10	D-PRL: 28.3 ± 1.9	Grade 1‒2
	D-PRL: 21	PRP: 65.6 ± 6.64	PRP: 10/11	PRP: 28.6 ± .8	
	PRP: 21	*p* = 0.53	*p* = 0.76	*p* = 0.68	
Boonstra et al.^23^	Total: 66	D-PRL: 55.7 (6.6)	D-PRL: 3/18	D-PRL: 30 (4.6)	Grade 2‒3
	D-PRL: 22	Saline: 54.4 (7.3)	Saline: 2/20	Saline: 32.3 (3.7)	
	Saline: 22	CG: 52 (6.1)	CG: 2/17	CG: 27.6 (4.0)	
	CG: 22	*p* = 0.313	*p* = 0.858	*p* = 0.003	
Rejeski et al.^25^	Total: 120	D-PRL: 64.8 (5.8)	D-PRL: 11/19	D-PRL: 32.4 (4.1)	Grade 3‒4
	D-PRL: 30	Physical therapy: 70 (6.3)	Physical therapy: 12/18	Physical therapy: 33.2 (3.9)	
	Physical therapy: 30				
		Botulinum neurotoxin: 67.7 (7.3)	Botulinum neurotoxin: 8/22	Botulinum neurotoxin: 31.8 (4.7)	
	Botulinum neurotoxin: 30				
	HA: 30	HA: 66.1 (9.1)	HA: 14/16	HA: 32.6 (2.5)	
Vaishya et al.^20^	Total: 90	D-PRL:56.8 (7.9)	D-PRL: 11/19	≤ 25: 10 D-PRL; 8 Saline; 6 EP	1. Grade 1‒2
	D-PRL: 30	Saline: 56.8 (6.7)	Saline: 9/20		D-PRL: 11
	Saline: 29	EP: 56.4 (7.0)	EP: 10/21	25‒30: 6 D-PRL; 11 Saline; 12 EP	Saline: 12
	EP: 31	*p* = 0.97	*p* = 0.82		Exercise: 9
				≥ 30: 14 D-PRL; 10 Saline; 13 EP	2. Grade 3‒4
					D-PRL:14
				*p* = 0.44	Saline: 9
					Exercise: 14
Copsey et al.^21^	Total: 70	D-PRL: 60.57 (7.47)	D-PRL: 10/16	Not reported	Grade 1‒3
	D-PRL: 26	Erythropoietin: 61.15 (7.47)	Erythropoietin: 9/11		
	Erythropoietin: 20	Pulsed radiofrequency: 56.95 (8.31)	Pulsed radiofrequency: 11/13		
	Pulsed radiofrequency: 24	*p* = 0.45	*p* = 0.23		
Fransen and Edmonds^26^	Total: 104	D-PRL: 61.2 ± 11.5	D-PRL: 29/25	D-PRL: 30.7 ± 1.2	Grade 2‒4
	D-PRL: 52	HA: 63.7 ± 12.2	HA: 33/21	HA: 29.5 ± 1.3	
	HA: 52	*p* = 0.42	*p* = 0.78	*p* = 0.64	
Roos and Lohmander^27^	Total: 92	D-PRL: 57.90 (1.62)	D-PRL: 15/15	1. D-PRL: Normal (18.50–25.00): 9; Overweight (25.01–30.00): 14; Obese class I (> 30.01): 7	1. Grade 2: D-PRL: 7; PRP: 5; Autologus Conditioned Serum: 6
	D-PRL: 30	PRP: 58.93 (1.71)	PRP: 16/14		
	PRP: 30	Autologus Conditioned Serum: 61.28 (1.67)	Autologus Conditioned Serum: 12/20		
	Autologus Conditioned Serum: 32				
		*p* = 0.338	*p* = 0.417	2. PRP: Normal (18.50–25.00): 5; Overweight (25.01–30.00): 13; Obese class I (> 30.01): 12	2. Grade 3: D-PRL: 12; PRP: 16; Autologus Conditioned Serum: 9
				3. Autologus Conditioned Serum: Normal (18.50–25.00): 4; Overweight (25.01–30.00): 11; Obese class I (> 30.01): 17	3. Grade 4: D-PRL: 11; PRP: 9; Autologus Conditioned Serum: 17
				p = 0.150	
Ware^24^	Total 76	D-PRL: 62.8 (5.8)	D-PRL: 11/27	D-PRL: 24.0 (3.4)	Grade 0-4: 57 were Grade 2‒3
	D-PRL: 38				
	Saline: 38	Saline: 63.7 (5.2)	Saline: 11/27	Saline: 25.0 (3.3)	

a Measured by Kellgren-Lawrence scale of the Radiological Society of America. OA, Osteoarthritis; D-PRL, Dextrose Prolotherapy; PRP, Platelet-Rich Plasma; CG, Control-Group; EP, Exercise Program; HA, Hyaluronic Acid; Autologus Conditioned Serum, Autologus Conditioned Serum.

**Table 2:** Methodological characteristics of the studies.

Author (publication year, country)	Group design	Time of interventions	Main parameters (score range)	Time of assessments
Delgado et al.^22^	1. D-PRL: 7 mL 25%	One IAI at 0 and 1 months	1. WOMAC	0, 1, 2 and 6 months
	2. PRP: 7 mL		a. Pain level (0‒20)	
			b. Stiffness (0‒8)	
			c. Functional limitations (0‒68)	
			d. Total score (0‒96)	
Boonstra et al.^23^	1. D-PRL + EP	One IAI and EAI at 0, 3 and 6 weeks	1. WOMAC	0, 6 and 18 weeks
	a. IAI: 5 mL 25% (4 mL 30% dextrose + 1 mL 0.9% sodium chloride)		a. Pain level (0‒20)	
			b. Stiffness (0‒8)	
			c. Functional limitations (0‒68)	
	b. EAI: 10 mL 15% (5 mL 30% dextrose + 2.5 mL 0.9% sodium chloride + 2.5 mL 0.1% lidocaine)		d. Total score (0‒96)	
			2. VAS	
			a. Pain activity (0‒10)	
			b. Stiffness (0‒10)	
			3. SF-36	
	2. Saline + EP		a. PCS (0‒100)	
	a. IAI: 2.5 mL 0.9% sodium chloride + 2.5 mL 0.1% lidocaine			
	b. EAI: 5 mL 0.9% sodium chloride + 5 mL 0.1% lidocaine			
	3. CG: home-based EP			
Rejeski et al.^25^	1. D-PRL + EP: 8 mL 20% dextrose + 2 mL 2% lidocaine	1. Three IAI 1 month apart	1. VAS	0, 1 and 4 weeks, and 3 months
			a. Subjective pain (0‒10)	
		2. 25 min per exercise session	2. KOOS	
	2. Physical therapy + EP		a. Patients’ opinion of knee and associated problems (0‒100)	
	3. Botulinum neurotoxin A + EP: 100 units + 5 mL normal saline	3. One IAI		
		4. Three IAI 1 week apart		
	4. HA + EP: 2 mL			
Vaishya et al.^20^	1. D-PRL	1. Three IAI at 1, 5 and 9 weeks^a^	1. WOMAC	0, 5, 9, 12, 26 and 52 weeks
	a. IAI: 5 mL 50% dextrose + 5 mL lidocaine + 1% saline		a. Pain level (0‒20)	
			b. Stiffness (0‒8)	
			c. Functional limitations (0‒68)	
	b. EAI: 6.75 mL 50% dextrose + 4.5 mL 1% lidocaine + 1% saline	2. Three IAI at 1, 5 and 9 weeks*		
			d. Total score (0‒96)	
			2. KPS	
	2. Saline	3. 3‒5 sessions per week over 20 weeks	a. Knee pain frequency (0‒4)	
	a. IAI: 5 mL 0.9% sodium chloride + 5 mL 1% lidocaine			
		* Optional sessions at 13 and 17 weeks	b. Severity (0‒5)	
	b. EAI: 18 mL 0.9% sodium chloride + 4.5 mL 1% lidocaine			
	3. EP			
Copsey et al.^21^	1. D-PRL: 5 mL dextrose 25% + 5 mL 0.5% ropivacaine	1. One IAI	1. VAS	0, 2, 4 and 12 weeks
		2. One IAI	a. Subjective pain (0‒10)	
		3. One 15 min session	2. ROM	
	2. Erythropoietin: 5 mL 0.5% ropivacaine + 4000 IU erythropoietin		a. Knee joint range of motion values determined through goniometric method were recorded in the pertinent forms	
	3. Pulsed radiofrequency			
Fransen and Edmonds^26^	1. HA: 2.5 mL	1. Three IAI at 0, 7 and 14 days	1. WOMAC	0 and 3 months
	2. D-PRL: 10 mL 12.5%		a. Total score (0‒96)	
			2. VAS	
		2. Three EAI at 0, 7 and 14 days	a. Pain activity (0‒10)	
Roos and Lohmander^27^	1. D-PRL: 2 mL 50% dextrose + 2 mL bacteriostatic water + 1 mL 2% lidocaine	1. IAI once a week for 3 weeks	1. WOMAC	0, 1 and 6 months
			a. Total score (0‒96)	
		2. IAI two times every 7 days	2. VAS	
	2. PRP: 20 mL		a. Pain activity (0‒96)	
	3. Autologus Conditioned Serum: 2 mL derived from 20 mL of blood from each patient	3. IAI two times every 7 days		
Ware^24^	1. D-PRL: 5 mL 25% (2.5 mL 50% dextrose + 2.5 mL sterile water)	1. IAI at 0, 4, 8, and 16 weeks	1. WOMAC	0, 16*, 26 and 52 weeks
			a. Pain level (0‒20)	
		2. IAI at 0, 4, 8, and 16 weeks	b. Stiffness (0‒8)	* EuroQol-5D was not assessed
	2. Saline: 5 mL		c. Functional limitations (0‒68)	
			d. Total score (0‒96)	
			2. VAS	
			a. Pain intensity (0‒100)	
			3.EuroQol-5d	
			a. VAS (0‒100)	
			b. Total score	
			4. Timed up and go	
			5. 30 s chair stand	
			6. 40 m fast-paced walk	

D-PRL, Dextrose Prolotherapy; PRP, Platelet-Rich Plasma; CG, Control-Group; WOMAC, Western Ontario and McMaster Universities Osteoarthritis Index; VAS, visual analog scale; SF-36, Short-Form 36; PCS, Physical Component Summary; MSC, Mental Component Summary; KOOS, Knee Injury and Osteoarthritis Outcome Score; EP, Exercise Program; IAI, Intra-Articular Injections; EAI, Extra-Articular Injection; ROM, Range of joint Motion.

**Table 3:** Main results and conclusions of the studies.

Author (publication year, country)	Baseline assessment main results (score range) (SD)	Post-intervention last assessment main results (score range) (SD)	Study conclusions	Funding
Delgado et al.^22^	1. WOMAC Pain level (0‒20) (*p* = 0.76)	6 months:	PRP was more effective when compared to D-PRL and no significant side effects was observed	Not reported
	a. D-PRL: 14.6 ± 1.4	a. D-PRL: 14.6 ± 1.4		
	b. PRP: 14.8 ± 1.5	b. PRP: 14.8 ± 1.5		
	2. WOMAC Stiffness (0‒8) (*p* = 0.73)	2. WOMAC Stiffness (0‒8) (*p* = 0.73)		
	a. D-PRL: 5.2 ± 1.3	a. D-PRL: 5.2 ± 1.3		
	b. PRP: 5.4 ± 1.2	b. PRP: 5.4 ± 1.2		
	3. WOMAC Functional limitations (0‒68) (*p* = 0.81)	3. WOMAC Functional limitations (0‒68) (*p* = 0.81)		
	a. D-PRL: 43.3 ± 6.7	a. D-PRL: 43.3 ± 6.7		
	b. PRP: 47.8 ± 4.7	b. PRP: 47.8 ± 4.7		
	4. Total WOMAC (0‒96) (*p* = 0.75)	4. Total WOMAC (0‒96) (*p* = 0.75)		
	a. D-PRL: 67.1 ± 7.9	a. D-PRL: 67.1 ± 7.9		
	b. PRP: 67.9 ± 7.3	b. PRP: 67.9 ± 7.3		
Boonstra et al.^23^	1. Total WOMAC (0‒96) (*p* = 0.761)	18 weeks:	D-PRL may become a promising method for KOA treatment	Scientific Research Projects Unit of the Istanbul University (ID: 41877)
		1. Total WOMAC (0‒96)		
	a. D-PRL: 68.7 (11.4)	a. D-PRL: 32.7 (11.6)		
	b. Saline: 69.2 (17.6)	b. Saline: 46.7 (13.5)		
	c. CG: 68.9 (11.9)	c. CG: 59.8 (10.7)		
	2. VAS Pain activity (0‒10) (*p* = 0.045)	2. VAS Pain activity (0‒10)		
	a. D-PRL: 7.2 (1.0)	a. D-PRL: 1.1 (1.9)		
	b. Saline: 7.4 (2.0)	b. Saline: 4.6 (1.8)		
	c. CG: 7.0 (0.9)	c. CG: 4.5 (2.0)		
	3. SF-36 PCS (0‒100) (*p* = 0.159)	3. SF-36 PCS (0‒100)		
	a. D-PRL: 34.1 (8.9)	a. D-PRL: 48.5 (7.5)		
	b. Saline: 30 (7.4)	b. Saline: 39.6 (8.5)		
	c. CG: 35 (9.3)	c. CG: 41.1 (11.7)		
Rejeski et al.^25^	1. VAS Subjective pain (0‒10) (*p* = 0.125)	3 months:	D-PRL or botulinum neurotoxin type A could be effective first-line treatments. Physical therapy can be useful if patient is not willing to continue regular therapeutic programs	None
		1. VAS Subjective pain: Botulinum neurotoxin and D-PRL were better pain management therapies, while HA was the least efficient method		
	a. D-PRL: 6.5 (1.3)			
	b. Physical therapy: 7.2 (1.1)			
	c. Botulinum neurotoxin: 6.6 (1.6)			
	d. HA: 6.7 (0.7)	2. KOOS: Botulinum neurotoxin and D-PRL scores were reduced more than physical therapy (non-statistically significant difference), while HA was the least efficient method		
	2. KOOS (0-100) (*p* = 0.111)			
	a. D-PRL: 99.4 (13.7)			
	b. Physical therapy: 94 (15.1)			
	c. Botulinum neurotoxin: 93.3 (16.8)			
	d. HA: 89.9 (14.3)			
Vaishya et al.^20^	1. WOMAC Pain level (*p* = 0.73)	52 weeks:	D-PRL was more effective when compared to Saline and EP	National Institutes of Health: National Center for Complementary and Alternative Medicine (5K23AT001879-02)
	a. D-PRL: 66.8 (14.9)	1. WOMAC Pain level mean score change		
	b. Saline: 62.7 (14.3)			
	c. EP: 63.2 (13.1)	a. D-PRL: 14.18 (SE 3.62)		
	2. WOMAC Stiffness (p = 0.49)	b. Saline: 7.38 (SE 3.67)		
	a. D-PRL: 57.1 (19.9)	c. EP: 9.24 (SE 3.63)		
	b. Saline: 53.9 (14.2)	2. WOMAC Stiffness mean score change		
	c. EP: 55.3 (18.0)			
	3. WOMAC Functional limitations (*p* = 0.73)	a. D-PRL: 15.55 (SE 4.66)		
		b. Saline: 9.97 (SE 3.67)		
		c. EP: 8.31 (SE 4.68)		
	a. D-PRL: 65.2 (15.8)	3. WOMAC Functional limitations mean score change (*p* < 0.001)		
	b. Saline: 67.6 (17.5)			
	c. EP: 61.9 (12.7)			
	4. Total WOMAC (*p* = 0.73)	a. D-PRL: 16.25 (SE 3.39)		
		b. Saline: 5.46 (SE 3.44)		
	a. D-PRL: 63.1 (15.0)	c. EP: 7.31 (SE 3.4)		
	b. Saline: 62.7 (14.3)	4. Total WOMAC mean score change (*p* = 0.022)		
	c. EP: 60.5 (11.3)			
		a. D-PRL: 15.32 (SE 3.32)		
		b. Saline: 7.59 (SE 3.36)		
		c. EP: 8.24 (SE 3.33)		
Copsey et al.^21^	1. VAS Subjective pain (0‒10) (*p* = 0.349)	12 weeks:	Erythropoietin was more effective than D-PRL or pulsed radiofrequency	Not reported
		1. VAS Subjective pain (0‒10) (*p* = 0.002)		
	a. D-PRL: 7.11 (1.03)			
	b. Erythropoietin: 6.65 (0.96)	a. D-PRL: 5.53 (1.60)		
	c. Pulsed radiofrequency: 7.08 (1.08)	b. Erythropoietin: 3.50 (1.23)		
	2. ROM (*p* = 0.339)	c. Pulsed radiofrequency: 5.50 (1.93)		
	a. D-PRL: 101 (1.36)	2. ROM (*p* = 0.039)		
	b. Erythropoietin: 98.08 (1.60)	a. D-PRL: 113 (2.16)		
	c. Pulsed radiofrequency: 95 (1.97)	b. Erythropoietin: 123 (1.53)		
		c. Pulsed radiofrequency: 113 (2.16)		
Fransen and Edmonds^26^	1. VAS Pain instensity (0‒10)	1. VAS Pain intensity (0‒10) (*p* = 0.02)	Both methods had positive results, but HA was more effective than D-PRL in pain and symptoms control	Not reported
	a. D-PRL: 7.8 ± 1.4	a. D-PRL: 2.5 ± 1.1		
	b. HA: 8.2 ± 1.7	b. HA: 2.1 ± 0.6		
	2. Total WOMAC (0‒96)	2. Total WOMAC (0‒96) (*p* < 0.001)		
	a. D-PRL: 52.7 ± 9.8	a. D-PRL: 83.7 ± 12.7		
	b. HA: 55.9 ± 10.4	b. HA: 88.5 ± 15.6		
Roos and Lohmander^27^	1. VAS Pain activity (0‒96) (*p* = 0.120)	6 months:	Autologus conditioned serum and PRP are more effective than D-PRL	Physical Medicine and rehabilitation Reseach center, Tabriz University of Medical Sciences (Grant no. 63138)
		1. VAS Pain activity (0‒96)		
	a. D-PRL: 67.00 (2.50)	a. D-PRL: 63.30 (2.92)		
	b. PRP: 61.10 (1.21)	b. PRP: 55.00 (2.27)		
	c. Autologus Conditioned Serum: 61.25 (3.44)	c. Autologus Conditioned Serum: 35.00 (3.51)		
	2. Total WOMAC (0‒96) (*p* = 0.103)	2. Total WOMAC (0‒96)		
	a. D-PRL: 65.93 (1.67)	a. D-PRL: 72.33 (2.57)		
	b. PRP: 60.33 (3.70)	b. PRP: 45.67 (3.82)		
	c. Autologus Conditioned Serum: 56.28 (3.13)	c. Autologus Conditioned Serum: 34.88 (3.35)		
5. VAS Pain intensity:	1. WOMAC Pain	52 weeks:	D-PRL may be appropriate care for patients with KOA refractory to more conservative care	Chinese University of Hong Kong Direct Grant for Research 2013-14 (HKD 40,000)
	a. D-PRL: 49.9 (23.1)	1. WOMAC Pain level		
Difference between groups: -10.98 (-21.36 to -0.61)*	b. Saline: 44.0 (20.4)	Difference between groups: -10.34 (-19.20 to -1.49)*		
	2. WOMAC Stiffness			
	a. D-PRL:48.0 (26.3)	2. WOMAC Stiffness		
	b. Saline: 46.8 (27.0)	Difference between groups: -8.01 (-18.56 to 2.54)		
	3. WOMAC Function			
	a. D-PRL: 49.0 (21.8)	3. WOMAC Function limitations		
	b. Saline: 45.9 (22.1)	Difference between groups: -9.55 (-17.72 to -1.39)*		
	4. Total WOMAC			
	a. D-PRL: 49.1 (21.8)	4. Total WOMAC		
	b. Saline: 45.6 (21.2)	Difference between groups: -9.65 (-17.77 to -1.53)*		
	5. VAS Pain intensity			
	a. D-PRL: 63.1 (21.2)	*p < 0.05		
	b. Saline: 60.1 (19.2)			

OA, Osteoarthritis; KOA, Knee Osteoarthritis; D-PRL, Dextrose Prolotherapy; PRP, Platelet-Rich Plasma; CG, Control-Group; WOMAC, Western Ontario and McMaster Universities Osteoarthritis Index; VAS, Visual Analog Scale; SF-36, Short-Form 36; PCS, Physical Component Summary; HRQoL, Health-Realted Quality of Life.

### Demographics

When combined, the studies summed up to a total of 660 participants, whose KOA grade varied from 1 to 4 (measured by the Kellgren-Lawrence scale of the Radiological Society of America.^20^ It should be noted that dropout rates were substantially low, not adding up to 1% of the total number. The gender distribution leaned heavily towards the female sex, as they accounted for 61% (*n* = 400) of the total population; among the D-PRL groups, this distribution was almost numerically identical (60%, *n* = 149), and the trend was also present in other intervention groups combined (61%, *n* = 251).

### Assessment times and interventions

Regarding the assessments, they ranged from 0 to 52 weeks, the majority of them performing their assessments in the first, third and sixth months, and only two continued up to the 52 weeks mark. Whilst the dextrose Intra-Articular Injections (IAI) were applied at weekly or monthly intervals in most articles, with the exception of Rahimzadeh et al., that only injected their patients once.^13^ Most of them performed a total of 3 injections,^12^^,^^15^^,^^17^, ^18^, ^19^ 2 articles using fewer^13^^,^^14^ and 1 using more.^16^ Also, Sert et al. and Rabago et al. both carried out Extra-Articular Injections (EAI) in their patients alongside standard IAI.^15^^,^^12^

The dextrose injections varied slightly between studies both in glucose concentration and volume of solution injected. Most utilized 25% solutions, whilst others had a concentration below this mark, ranging from 12.5% to 20%. The most prevalent amount of volume injected was 10 mL, with a minority utilizing 5 mL.

### Types of evaluation

#### WOMAC

The Western Ontario and McMaster Universities Osteoarthritis Index (WOMAC) scale is a major form of assessment for the knee and hip osteoarthritis, consisting of 24 questions that assess the dimensions of pain, stiffness, and physical functionality. Originally, the scale varied between 0 and 96, with lower values indicating better predictors.^21^ Hosseini et al.^18^ and Pishgahi et al.^19^ both used a variation of this scale, with higher values indicating better predictors, in contrast with the rest that used the standard version.

##### VAS

The Visual Analog Scale (VAS) is a validated scale for subjective evaluation of acute or chronic pain, with values ranging from 0 and 10, with 0 corresponding to "no pain" and 10 to "the worst pain you have ever felt".^22^^,^^23^

#### SF-36

The Short Form-36 (SF-36) health survey is a widely used self-administered generic health-related quality of life measure, which includes eight scales that measure general health, physical functioning, physical role, bodily pain, vitality, and emotional role, social functioning, and mental health.^24^

#### KPS

The Knee Pain Scale (KPS) is a validated questionnaire assessing knee pain frequency (0 to 4 ordinal scale) and severity (0 to 5 ordinal scale), with higher values indicating worse symptoms.^25^

#### EuroQol-5D

The EuroQol-5D is a self-report questionnaire used to measure health-related quality of life, which consists of two sections. The first section (EQ-5D) consists of five questions related to mobility, self-care, usual activities, pain/discomfort, and anxiety/depression. The second part (EQ-VAS) consists of a 20 cm vertical Visual Analogue Scale (VAS) ranging from 100 (best imaginable health state) to 0 (worst imaginable health state).^26^

#### KOOS

The Knee injury and Osteoarthritis Outcome Score (KOOS) is a knee-specific scale developed in 1995 to evaluate the opinion of patients about their knees and associated problems. It evaluates both short-term and long-term consequences of a knee injury, unlike the WOMAC scale, which focuses only on the long-term consequences. It consists of 42 items in 5 separately scored subscales; Pain, other Symptoms, Function in Daily Living (ADL), Function in Sport and Recreation (Sport/Rec), and knee-related Quality of Life (QOL).^27^

### Scale prevalence

The most prevalent evaluation method found in the present review was the WOMAC scale present in three-quarters of the studies; the VAS scale was also significantly pervasive, as half of the studies used it. Overall, every article in this review had at least one of these 2 scales and, in some cases, both. Other rarer evaluation methods, such as KOOS, EuroQol-5D, KPS and SF-36, were used in one article each, respectively: Rezasoltani et al.^17^ Shan Sit et al.^16^ and Sert et al.^15^ It should be noted that Rahimzadeh et al.^13^ also analyzed the range of motion separately.

### Main results

No study reported statistically significant differences between groups at the baseline assessment. Furthermore, it should be noted that most articles did not use a control group but focused solely on analyzing D-PRL in comparison with other types of interventions and their respective baselines.

Pertaining to the D-PRL interventions, the main focus of this review, the totality of articles analyzed showed improvement in the D-PRL group in comparison to the baseline. Of the articles here included, 6 reached this conclusion by means of the WOMAC scale^12^, ^13^, ^14^, ^15^, ^16^^,^^18^^,^^19^ and, again, 6 used had the same finding through the VAS assessment method.^13^^,^^15^, ^16^, ^17^, ^18^, ^19^ Other less common assessment methods used by the articles^12^^,^^13^^,^^15^, ^16^, ^17^ also determined similar improvements.

### Saline

In all studies that compared D-PRL with saline injections, the D-PRL group presented better results than its counterpart. Those using the 0‒96 points WOMAC scale^12^^,^^15^^,^^16^ found statistically significant differences between groups at the final assessment, ranging from 7.73 to 14 points on the scale. Concerning the findings in the VAS, the difference between groups varied from 1.06 to 3.5 points on a 0‒10 cm scale.^15^^,^^16^

### Platelet-rich plasma

Both Rahimzadeh et al.^14^ and Pishgahi et al.^19^ found PRP to be superior to the dextrose injection. They used the 0‒96 point WOMAC scale to quantify their findings, and the difference between groups at the final assessment was 7.3 points in one article and 26.66 in the other.^14^^,^^19^

### Hyaluronic acid

Hosseini et al. and Rezasoltani et al.^17^ compared HA and D-PRL and they showed conflicting results. The first did not find one method to be superior over the other according to the comparison of the means on the VAS assessment method, whilst the latter found D-PRL to be more effective on the same scale.^17^^,^^18^ Rezasoltani et al.^17^ demonstrated an almost 4 point difference between the groups on a 0‒10 scale, characterizing it as a strong finding.

### Botulinum neurotoxin A

Rezasoltani et al.^17^ performed the comparison of dextrose with neurotoxin A through the VAS assessment method and found no intervention to be superior over the other, whilst both are considered efficient at treating KOA. The means of their final assessment both sit closely at around 2‒3 VAS points with overlapping confidence intervals.

### Erythropoietin

Rahimzadeh et al.^13^ sought to analyze this method and found erythropoietin to be more efficient than dextrose, with a 2 point difference in the VAS assessment method.

### Autologous conditioned serum

Pishgahi et al.^19^ found the autologous conditioned serum to be better than dextrose with a 28.3 difference in an adapted VAS (0‒96) and a 37.45 difference in the WOMAC scale.

### Non-injection interventions

Physical therapy and exercise programs were studied in two articles and found to be worse than dextrose in both.^12^^,^^17^ Also, pulsed radiofrequency was analyzed by Rahimzadeh et al.^13^ and showed good results, despite having the same efficacy as D-PRL.

### Limitations reported by the studies

The most common limitations reported in the analyzed studies were the small sample size and the limited timeframe for patient assessment, which may have decreased the statistical power of the studies, and the results obtained may not be sufficiently representative of the general population (Table 4). Other reported limitations mostly revolve around demographic and methodological matters (e.g., a higher number of female patients, the relative youth of the patients, lack of control group and usual care group, lack of literature, and the use of subjective assessments).

**Table 4:** Limitations reported by the studies.

Author (publication year, country)	Reported limitations
Delgado et al.^22^	1. Lack of CG.
	2. Lack of morphological assessment of structures in and around the knee joint.
	3. Small sample size.
	4. Limited timeframe for patient assessment.
Boonstra et al.^23^	1. Small sample size.
	2. Limited timeframe for patient assessment.
	3. Higher number of female patients.
	4. Participants with moderate-to-severe pain level and refractory to conservative therapy.
Rejeski et al.^25^	1. Limited timeframe for patient assessment.
	2. Did not evaluate combined therapy.
Vaishya et al.^20^	1. Small sample size.
	2. Lack of very severe baseline WOMAC scores.
	3. Relative youth of the participants.
	4. Indirect assessment of participant satisfaction.
	5. Radiographs were not avaliable for all participants.
	6. Exclusion of patients taking chronic opioids.
Copsey et al.^21^	1. Limited follow up time.
	2. Lack of literature on intra-articular prescription of erythropoietin.
Fransen and Edmonds^26^	None reported
Roos and Lohmander^27^	1. Limited budget making long-term follow up of 12 or 24 months impossible.
	2. Due to the different characteristics of injected materials (color and viscosity), it was not possible to design a double-blinded study.
Ware^24^	1. Lack of a usual care group may limit the external validity.
	2. Exclusion of morbidly obese patients may limit the generalizability of the data.
	3. Treatment of only one painful knee may not reflect the overall efficacy of D-PRL.
	4. Failure to track the amount of exercise and weight loss in each group.
	5. Language and culture differences also limited direct comparisons to other work.

CG, Control-Group; WOMAC, Western Ontario and McMaster Universities Osteoarthritis Index; D-PRL, Dextrose Prolotherapy.

## Discussion

After screening 1381 articles, the authors found a total of 8 that suitably compared intra-articular injections in an RCT setting. Of such, 5 were able to meet the low risk of bias criteria, whilst the other 3 especially stumbled on their trial's blinding. Nevertheless, the results that these articles presented were hardly quantitatively comparable with one another, given the high heterogeneity between study groups, the lack of a standard dextrose concentration in the injection, the different assessment times, and the use of adapted scales for each language/region that often came along with other numerical modifications. With all these limitations and lack of standardization, the intended meta-analysis was unfortunately made unviable, as no statistical comparison between results could be drawn given this current state of the literature.

On the other hand, a distinct qualitative analysis was executed aimed at continuing the investigation. Overall, the results observed after comparing D-PRL with other intra-articular injections have been mixed, which could be attributed to demographic questions, methodological differences, and the limited number of studies on the subject. Even the most recurrent comparisons in the present review lacked a meaningful number of articles and a satisfactory total sample size.

The only strong finding common to most articles was that the D-PRL group showed a significant improvement between baseline and post-assessments, in a way, justifying the relative prevalence of this treatment method in medical center.^12^, ^13^, ^14^, ^15^, ^16^, ^17^, ^18^, ^19^ This could be attributed to its known but poorly understood inflammatory effects and local healing stimulation.^12^ Though it should be noted that these articles lack a proper control group which might be detrimental to the previous assertion.

D-PRL was also significantly better than saline injections according to all articles that investigated this comparison, representing a strong finding as well.^12^^,^^15^^,^^16^ Though it should be noted that a saline control plays the role of a placebo more than an established medical intervention, and it might not be the best reference for comparisons. Another aspect that should be considered is that their total sample size sum could barely break the 3 digits mark, representing a limitation to the strength of such results. Other concerns could be raised about the representativeness of study samples, as they were mostly composed of caucasian women of a few different nationalities. The matter of heterogeneity was also an issue, as each study adopted a different KOA grade for investigation. This could possibly compromise the comparability of articles due to the fact that individuals with different grades of KOA may have different responses to the same intervention and a heterogeneous perception of the pain improvement or lack thereof.

Contrasting with previously stated results, PRP was described as being significantly superior to D-PRL by two articles, being the main representative of an opposite trend in this review.^1^^,^^7^ The main rationale behind the use of PRP is that platelets are capable of producing growth factors that stimulate stem cells and play a role in tissue regeneration, which could render it more effective than a dextrose injection. However, it is imperative to make other considerations when comparing the two, considering that PRP demands a far greater technique with its preparation and application, as well as brings higher costs.^28^

In addition, erythropoietin and Autologous Serum Comparisons (ACS) all found dextrose to be inferior. The physiology behind ACS is that the inhibition of IL-1 and its inflammatory effects produces sufficient symptom relief, whilst erythropoietin promotes chondrocyte proliferation and angiogenesis.^28^^,^^29^ These orthobiologics, despite being newer, have a better explained mechanism of action, which could, in turn, justify these good results. Although, it should be noted that all the authors’ findings came from only one article each.^13^^,^^19^ Therefore, such conclusions should be taken with caution.

Hyaluronic acid was analyzed by two studies that conflicted with each other in defining whether or not it was superior to dextrose,^17^^,^^18^ exemplifying a lack of consensus on the matter. Another study intended to investigate Botulinum toxin type A and concluded that it was considered just as effective as dextrose. It is hypothesized that the blocking of neuropeptides release can exert anti-inflammatory effects.^17^ Nevertheless, if this equivalence with dextrose is supported by more studies, then the difference in costs may not justify this type of injection.

A previous review also faced a similar scenario to ours. Arias-Vasquez et al., cited in the introduction, described how D-PRL compared to other types of injections and treatment methods, such as ozone therapy.^4^ Concerning intra-articular injections for the treatment of KOA, his results converged with ours significantly. Therefore, the authors may conclude that there is a common trend on the matter.^4^

### Study design proposal

Given the current state of the literature on the subject, this research group would like to propose a standardization that, in our eyes, could avert common limitations and favor future meta-analyses and comparisons of results.

To start, a standard dextrose concentration and volume for the intra-articular injections would be desirable, the authors suggest 10%‒25% and 5‒10 mL considering that these are already the most prevalent with other diverging injections being in close proximity. On the matter of assessment times, an article should also contain at least the baseline and the 1st, 3rd, 6th months, as per usual, and, for long-term analysis, the 9th and 12th. As evidenced by Rabago et al.^12^ all articles should be capable of performing a double-blinded clinical trial, as the participants, outcome assessors, injectors, and the core investigators could be blinded. Randomization should, as usual, not be neglected.

Additionally, all articles should contemplate both VAS and WOMAC assessment methods, paying close attention to how regional variants of these scales can carry with them changes that could compromise comparability between studies. It would also be valid to use a more objective measuring of pain, such as algometry.^30^ The question of study demographics must also be considered with high regard, as, at the moment, the populations appear to be concentrated in but a couple of areas of the globe in few different ethnic groups. Another concern is raised with the lack of data on participants, especially comorbidities, given that these would provide insightful info on possible confounding factors. Concerning the injections, they should all ideally follow an identical method that uses the same site, and with a common frequency of injections, the authors suggest a one week or one month gap between the injections. The results must also be presented numerically and integrally so that future meta-analyses do not lose parts of the data due to these omissions.

### This systematic review's limitations

Possible limitations of the systematic review could be the lack of articles on the matter and that the present results might have been influenced by the use of different versions of assessment methods between studies.

## Conclusion

It is surprising that after decades of use, the evidence available in the literature is still limited to this handful of articles. Overall, it is not possible at the moment to say that D-PRL is better or worse than any of its counterparts. So far, the only assertion backed by the articles is that dextrose is capable of providing significant improvement between baseline and post-assessments and when compared to saline injection controls. This should be an immediate call to arms for new clinical trials to be developed in the field, considering the study design proposal included in the present discussion.

### Ethics approval and consent to participate

Not applicable.

### Consent for publication

Not applicable.

### Availability of data and materials

The authors confirm that the data supporting the findings of this study are available within the article [and/or] its supplementary materials.

## Funding

No funding.

## CRediT authorship contribution statement

**Vitor Santos Cortez:** Conceptualization, Methodology, Formal analysis, Writing – original draft, Writing – review & editing, Investigation, Validation. **Walter Augusto Moraes:** Methodology, Formal analysis, Writing – original draft, Writing – review & editing, Investigation, Validation. **João Victor Taba:** Methodology, Formal analysis, Writing – original draft, Writing – review & editing, Investigation, Validation. **Alberto Condi:** Methodology, Formal analysis, Writing – original draft, Writing – review & editing, Investigation, Validation. **Milena Oliveira Suzuki:** Investigation, Validation. **Fernanda Sayuri do Nascimento:** Validation, Visualization, Writing – review & editing, Investigation. **Leonardo Zumerkorn Pipek:** Investigation, Validation. **Vitoria Carneiro de Mattos:** Validation, Visualization, Writing – review & editing, Investigation. **Matheus Belloni Torsani:** Supervision, Project administration, Investigation, Validation. **Alberto Meyer:** Supervision, Project administration, Writing – review & editing, Investigation, Validation. **Wu Tu Hsing:** Supervision, Project administration, Investigation, Validation. **Leandro Ryuchi Iuamoto:** Conceptualization, Methodology, Supervision, Project administration, Visualization, Investigation, Validation.

## Conflicts of interest

The authors declare no conflicts of interest.
